# Update on the Diagnostic Pitfalls of Autopsy and Post-Mortem Genetic Testing in Cardiomyopathies

**DOI:** 10.3390/ijms22084124

**Published:** 2021-04-16

**Authors:** Simone Grassi, Oscar Campuzano, Mònica Coll, Francesca Cazzato, Georgia Sarquella-Brugada, Riccardo Rossi, Vincenzo Arena, Josep Brugada, Ramon Brugada, Antonio Oliva

**Affiliations:** 1 Department of Health Surveillance and Bioethics, Section of Legal Medicine, Fondazione Policlinico A. Gemelli IRCCS, Università Cattolica del Sacro Cuore, 00168 Rome, Italy; francescacazzato993@gmail.com (F.C.); riccardo.rossi@unicatt.it (R.R.); antonio.oliva@unicatt.it (A.O.); 2 Centro de Investigación Biomédica en Red de Enfermedades Cardiovasculares (CIBERCV), 28029 Madrid, Spain; oscar@brugada.org (O.C.); mcoll@gencardio.com (M.C.); jbrugada@clinic.cat (J.B.); ramon@brugada.org (R.B.); 3 Cardiovascular Genetics Center, Institut d’Investigació Biomèdica Girona (IDIBGI), University of Girona, 17190 Girona, Spain; 4 Medical Science Department, School of Medicine, University of Girona, 17003 Girona, Spain; georgia@brugada.org; 5 Arrhythmias Unit, Hospital Sant Joan de Déu, University of Barcelona, 08950 Barcelona, Spain; 6 Area of Pathology, Department of Woman and Child Health and Public Health, Fondazione Policlinico Universitario A. Gemelli IRCCS, 00147 Rome, Italy; Vincenzo.arena@policlinicogemelli.it; 7 Istituto di Anatomia Patologica, Università Cattolica del Sacro Cuore, 00168 Rome, Italy; 8 Institut Clínic Cardiovascular (ICCV), Hospital Clínic, Universitat de Barcelona, Institut d’Investigacions Biomèdiques August Pi i Sunyer (IDIBAPS), 08036 Barcelona, Spain

**Keywords:** cardiomyopathies, sudden cardiac death, forensic autopsy, post-mortem genetic testing, virtopsy

## Abstract

Inherited cardiomyopathies are frequent causes of sudden cardiac death (SCD), especially in young patients. Despite at the autopsy they usually have distinctive microscopic and/or macroscopic diagnostic features, their phenotypes may be mild or ambiguous, possibly leading to misdiagnoses or missed diagnoses. In this review, the main differential diagnoses of hypertrophic cardiomyopathy (e.g., athlete’s heart, idiopathic left ventricular hypertrophy), arrhythmogenic cardiomyopathy (e.g., adipositas cordis, myocarditis) and dilated cardiomyopathy (e.g., acquired forms of dilated cardiomyopathy, left ventricular noncompaction) are discussed. Moreover, the diagnostic issues in SCD victims affected by phenotype-negative hypertrophic cardiomyopathy and the relationship between myocardial bridging and hypertrophic cardiomyopathy are analyzed. Finally, the applications/limits of virtopsy and post-mortem genetic testing in this field are discussed, with particular attention to the issues related to the assessment of the significance of the genetic variants.

## 1. Introduction

Sudden unexplained death (SUD) is a fatal event that encompasses several heart disorders which lead to abrupt and unpredicted death. Normally, the victim has no known history of heart disease. In adult population (16–64 years) the SUD rate is 11/100,000 per year, while, in the young population (<16 years of age), it is 7.5/100,000 [1]. According to the evidence of the last 15 years, most of the SUD cases (at least the 5–20% of them) are of cardiac origin [2]. It is well known that Sudden Cardiac Death (SCD) is one of the most common causes of death in developed countries, with a yearly incidence of 30–200/100,000. In young population, SCD is a rare event, having an incidence of approximately 2–5/100,000 patients per year [3]. Coronary artery disease and acquired cardiomyopathies are the most frequent causes of SCD in the adults, while in those younger than 35 years the main cause of SCD is represented by non-ischemic diseases [4,5]. Cardiomyopathies are the main cause of SCD in those younger than 35 years, while up to 40% of young cases of SCD are caused by pathogenic alterations in the genes that code for ion channels or proteins associated with their proper functioning [6,7]. Currently, nearly the 20% of total deaths in young population remain without a conclusive explanation after a complete autopsy [8,9,10,11]. Inherited arrhythmogenic syndromes—channelopathies—account for most of the autopsy-negative cases (if acute intoxications are excluded). On the other hand, cardiomyopathies are generally thought to have distinctive macroscopic and microscopic features. However, in the forensic field cardiomyopathies are often extremely challenging, mainly because of two factors: (i) the phenotypes of cardiomyopathies gradually develop, and some of cases of SCD occur in young victims, with only mild microscopic and/or macroscopic signs of disease; (ii) when the diagnosis has not been made before the death, at the autopsy it is often difficult to distinguish a cardiomyopathy from another pathological (or from a physiological) condition.

In this paper, we review the main issues that the pathologist can encounter at the macroscopic and microscopic examination of cardiomyopathies cases and we discuss the contributions that can be given by virtopsy and post-mortem genetic testing, focusing on the issues of medico-legal interest.

## 2. Hypertrophic Cardiomyopathy

**Figure 1:**
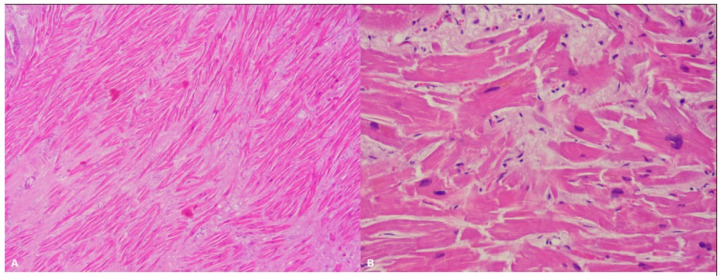
Left ventricle: diffuse disarray and mild fibrosis (**A**). Hematoxylin and eosin stain, 4× magnification), associated with focal and mild myocardial hypertrophy (**B**). Hematoxylin and eosin stain, 40× magnification) in a 48-year-old man who suddenly died while playing sport.

Hypertrophic cardiomyopathy (HCM) has a prevalence of 1/500 [12]. It is macroscopically characterized by abnormal left ventricular (LV) wall thickness and/or heart weight. In HCM the LV hypertrophy is typically asymmetrical, but it can also be symmetrical and can involve only delimited regions, like the apex. The LV wall thickness is considered a strong predictor of sudden death [13]. However, the predisposition to SCD is multifactorial, since it also depends on anamnestic factors (familiarity for SCD and history of syncope and/or non-sustained ventricular tachycardia) [14]. At the autopsy, as said, an abnormal heart weight can be suggestive of HCM, but there is little evidence on what the normal range of this parameter is [15]. However, it is usually assumed that a weight > 500 g is of pathological significance [16]. The diagnostic microscopic features are hypertrophy and disarray (i.e., the loss of the normal parallel alignment of the myocytes), associated with interstitial fibrosis (Figure 1). LV hypertrophy and/or myocardial fibrosis without myocardial disarray are features of uncertain significance [17].

### 2.1. Differential Diagnosis

**Figure 2:**
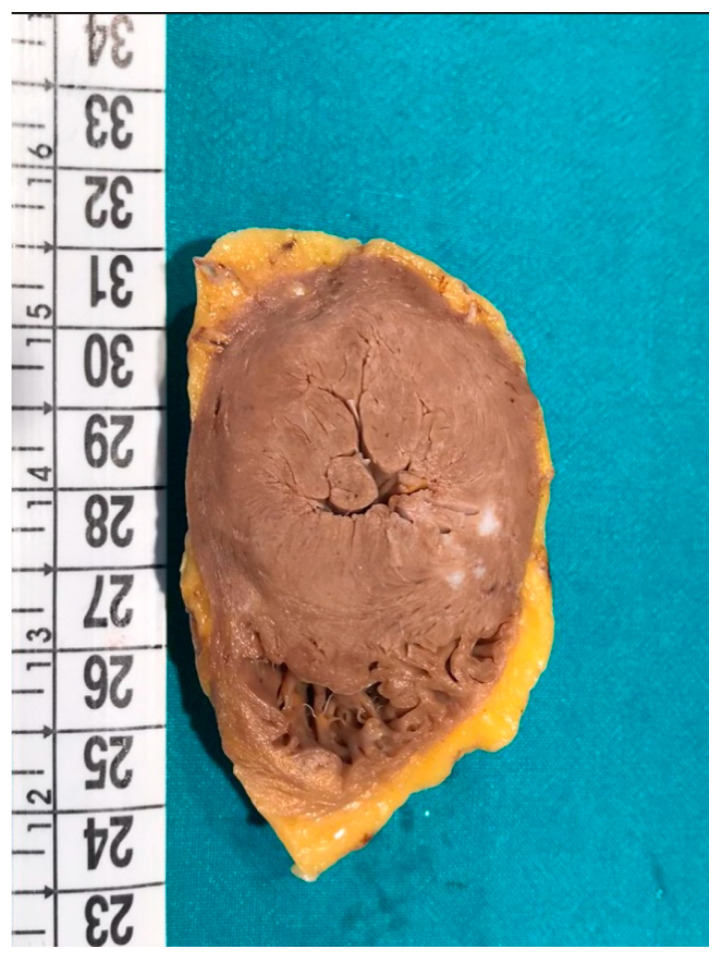
Macroscopic transversal section of the heart of a 48-year-old man affected by hypertrophic cardiomyopathy (HCM) (the same case of Figure 1). Concentric hypertrophy is clearly visible.

At the autopsy, the hypertrophy of the ventricular walls is the most misleading feature: ventricular hypertrophy is an extremely common finding, but it must be carefully evaluated to infer on its origin. In detail, the hypertrophy can be concentric or eccentric. Concentric hypertrophy may be associated with both congenital (HCM) (Figure 2) and acquired (hypertension, aortic stenosis, chronic abuse of anabolic steroids) conditions. Eccentric hypertrophy is rarely found at the autopsy, since it does not reduce the volumes of ventricular chambers, and thus the diagnosis can be easily missed. Eccentric hypertrophy is usually caused by valvular anomalies, like aortic or mitral insufficiency.

Furthermore, several adaptive or idiopathic conditions can be misdiagnosed as HCM. For instance, in obese individuals a state of chronically high cardiac output can cause the so-called “obesity associated heart disease”, that can mimic HCM [18]. Moreover, competitive athletes (in particular endurance athletes) frequently show an adaptive ventricular remodelling known as athletic heart syndrome (“athlete’s heart”). Because of the small increases in the thickness of the ventricular walls, athlete’s heart can be easily misdiagnosed as a mild HCM (a LV wall of 13–15 mm is typically considered a diagnostic grey zone) [19]. However, differently from HCM, athlete’s heart is usually associated with left atrium enlargement and, according to current evidence, does not increase the risk of supraventricular tachyarrhythmias [20,21]. Finally, in the young population (in both athletes and non-athletes) an idiopathic left ventricular hypertrophy (ILVH) can be reported [22]. The ILVH is a condition of uncertain significance, despite it has been associated with an increased risk of sudden death, especially in presence of myocardial fibrosis [17].

### 2.2. Diagnostic Issues in Young SCD Victims

In HCM the impaired mechanical function of myocytes progressively causes compensatory disarray, hypertrophy, and fibrosis. Therefore, in children this cardiomyopathy often has no or extremely mild phenotype, with most of the patients developing clear hypertrophy only during or after the adolescence [22,23]. In case of young victims, since hypertrophy is rarely found, it is very important to look for the myocyte disarray. This finding can be physiological in the parts of the anterior and posterior walls of the right ventricle that are near the septum, and thus it is very important to collect several samples in different areas of the heart [23].

HCM is characterized by variable expressivity, and thus a carrier of a pathogenic variant could never develop macroscopic or microscopic signs of disease. However, genotype-positive patients without hypertrophy of the LV walls still have altered cardiac dimensions/function and a higher burden of early phenotypes [22].

### 2.3. HCM and Myocardial Bridging

**Figure 3:**
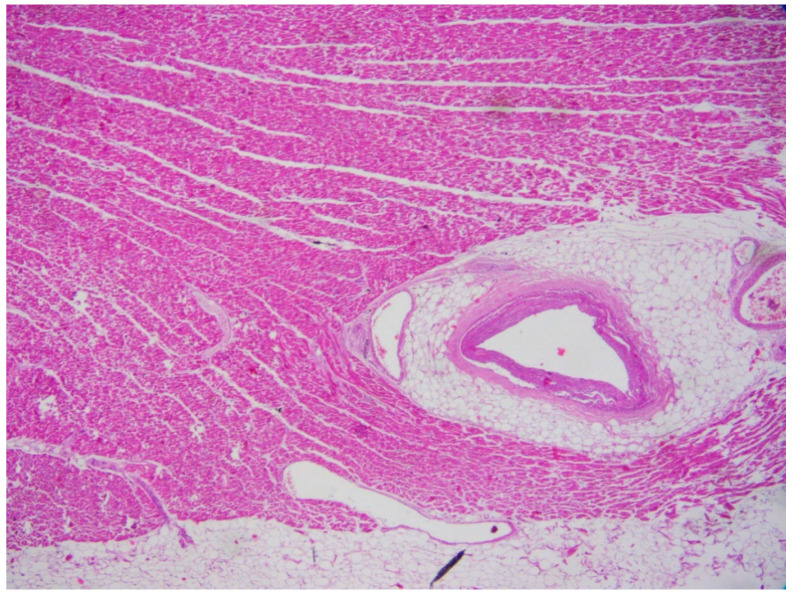
Myocardial bridging in the heart of a 32-year-old man who suddenly died (Hematoxylin and eosin stain, 20× magnification).

Myocardial bridging (MB) (Figure 3) is a common congenital anomaly, that can be found in up to the 85% forensic autopsies. It usually affects the middle trait of the left anterior descending coronary artery [24,25].

Brodsky et al. found that variables like age, sex, body mass index, heart weight, left ventricle wall thickness, and circumference of pulmonary/aortic valves do not have a statistically significant relationship with MB [26].

MB is more frequently reported in forensic rather than in clinical scientific literature: according to current evidence, it is found in up to the 16% of coronary angiographies and in up to the 85% of forensic autopsies [26].

It is a condition of uncertain clinical significance. However, despite very common, some authors have reported associations between MB and myocardial ischemia, left ventricle dysfunction, arrhythmias, and sudden cardiac death [6,27,28]. In particular, Schwarz et al. found that MB can cause a decrease in coronary flow reserve, which may be responsible for myocardial ischemia [29]. Moreover, Mohiddin et al., found that thallium perfusion is reduced by the LV hypertrophy of the area where the MB is, but they did not find any significant association between this vascular anomaly and ventricular arrhythmias or sudden death [30]. Despite some cases of SCD in young patients with MB have been reported, the first clinical manifestation generally occurs in the adult age, and it is thought to be related to a loss of elasticity of the coronary arteries (and thus to a reduction of diastolic lumen) [6,23,31].

From a forensic point of view, one of the most interesting issues regarding MB is its possible association with some features of HCM or with HCM itself [6,32,33,34,35]. Several authors reported that the depth of the intramyocardial course (≥2 mm) is significatively associated with a greater amount of myocardial fibrosis [28,32,36]. The fibrotic tissue is due to an ischemic process in the territory of the MB, which consequently could predispose the individual to an increased risk of sudden death [36,37]. As noted by Yetman et al., chronic ischemia can cause a myocardial damage characterized by disarray and diffuse fibrosis, which can consequently predispose the subject to develop ventricular arrhythmias and sudden death [32]. In general, the prevalence of MB among patients with HCM has been reported to be of 21–41% [33]. This association is considered important by many authors because the pathological substrate of HCM can increase the risk of MB clinical manifestation. For example, Sharzhee et al. observed that the structure of MB is essentially the same in HCM and non-HCM individuals, but the heart hypertrophy causes a greater compression of the bridging, which is responsible for the decrease in the flow rate and for a higher pressure drop coefficient [38].

### 2.4. Genetics

HCM normally has an autosomal dominant inheritance pattern, and it is usually caused by rare variants in genes encoding cardiac sarcomeric proteins [39,40,41]. Variants in two genes (*MYH7* and *MYBPC3*) account for 60–70% of HCM patients with known pathogenic variants, while pathogenic variants of *TNNI3*, *TNNT3*, *TPM1* and *MYL3* are rarer [42,43,44]. Rare variants in the *MYH7* gene are predominantly missense, so single nucleotide-base variants result in a non-synonymous single amino acid substitution [45,46]. The majority of rare alterations in the *MYBPC3* gene are caused by the introduction of a premature stop codon (PTC), that results in a truncated protein transcript. This PTC may be caused by nonsense mutations and insertions/deletions that alter the reading frame (known as frameshifts) or splice-site variants [43,47].

HCM can also (rarely) be caused by mutations of non-sarcomeric genes, like *CSRP3* (coding for a Z-disk protein), *FHL-1* (coding for a sarcomere-associated protein) and *PLN* (coding for a regulator of sarcoplasmic reticulum calcium) [48,49].

The penetrance of the pathogenic variants is variable, being relatively high for some of them (in particular, *MYH7* and *MYBPC3)* and low to moderate for many others [49]. Moreover, as said, HCM shows a significant variability in its phenotypic expression. Indeed, the severity of the phenotype (and thus the risk of SCD) is thought to be increased, for example, by an insertion/deletion variant in the angiotensin-1 converting enzyme gene *(ACE*) and by relevant changes in loading conditions (like systemic arterial hypertension) [48]. Moreover, in the carriers of pathogenic sarcomere protein mutations, hypertrophy and myocardial fibrosis tend to be more severe and the prognosis is generally poorer than in patients in which no variant is found [50]. In particular, the phenotype is more severe in those who carry multiple variants (about the 5% of the total carriers) [50].

Finally, it should be noted that mutations of sarcomeric genes like *MYH7* or *TNNT2* may have pleiotropic effects, causing different cardiomyopathies within the same families [48].

## 3. Arrhythmogenic Cardiomyopathy

Arrhythmogenic Cardiomyopathy (ACM) has a prevalence up to 1/2000 [51], being particularly frequent in some geographical areas like Veneto region (Italy). It is characterized by the fibrofatty replacement of the myocardium, that progresses from the epicardium towards endocardium and tends to be in the “triangle of dysplasia” (outflow tract of right ventricle, inferior wall beneath the posterior tricuspid leaflet and the ventricular apex) [52]. Epicardial adiposity is supposed to have arrhythmogenic effects through different patterns of action: structural barrier to electric impulse propagation, adipogenesis/fibrosis, increased oxidative stress, and formation of cytokines [53]. However, the clinical significance of this recurrent finding is still to be determined.

### 3.1. Differential Diagnosis

**Figure 4:**
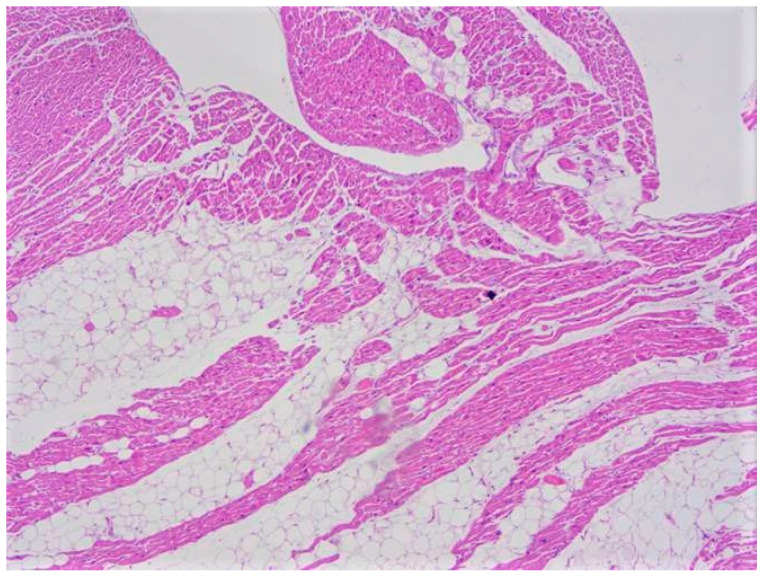
Fatty infiltration with an epicardial-endocardial gradient clearly separated by the myocardium and without fibrosis (adipositas cordis). This case regards an obese 22-year-old woman who suddenly died at home (hematoxylin and eosin stain, 10× magnification).

Fibrofatty replacement is not pathognomonic of ACM, since it can also be found, for example, in *PRKAG2* cardiac syndrome, Uhl’s anomaly, and HCM [54,55]. However, the most important differential diagnosis is the normal (and sporadic) fatty infiltration of the ventricles (i.e., “adipositas cordis”), that is often found (up to the 85% of the cases), especially in the elderlies and in the obese patients (Figure 4) [56,57]. Corradi et al., observed that a normal heart contains an average amount of 205 g of ventricular myocardium and 54 g of ventricular fat [58]. In normal hearts, a certain amount of subepicardial fatty tissue is often found in the ventricular walls (especially in the antero-lateral and apical areas), but it is clearly separated from the inner myocardium [33,59]. Some authors, like Anumonwo et al., reported that the volume and the thickness of epicardial fat are possible markers of ACM, considering pathological a volume greater than 125 mL and a thickness greater than 5 mm [53]. However, normal fatty infiltration can be easily distinguished from ACM through the observation of myocytes atrophy, that gives the ventricular wall a translucent appearance at the autopsy. Another important differential diagnosis is myocarditis, since in ACM signs of recurrent, chronic myocarditis (inflammatory cells infiltrates with focal myocyte necrosis) can often be observed [33].

### 3.2. Genetics

ACM can be caused by deleterious alterations located in genes encoding mainly desmosomal proteins but also proteins involved in electric signal transmission [60,61,62,63,64]. Currently, more than 1000 rare genetic variants have been identified in more than 15 genes (*ANK2, CTNNA3, DES, DSC2, DSG2, DSP, FLNC, ILK, JUP, LMNA, PKP2, PLN, PNPLA2, SCN5A, TGFB3, TJP1, TMEM43, TP63,* and *TTN*), but only about 400 rare genetic alterations have been classified as certainly pathogenic [65]. All the other rare variants have an ambiguous role and further data are needed to determine whether they are pathogenic for ACM or not [66]. Most of the pathogenic variants affect desmosomal genes, like *PKP2* (41.6% of the pathogenic variants), *DSP* (21.2%), *DSG2* (12.2%), *DSC2* (9.7%), and *JUP* (3.6%) [65]. In up to the 16% of the cases, a single patient carries multiple mutations, that can affect the same gene (compound heterozygosity) or different genes (digenic heterozygosity) [65,67,68]. Furthermore, ACM can be caused by variants of extradesmosomal genes, like *LMNA*, *DES* and *TTN* [65].

When a carrier of a pathogenic variant is found, his relatives should be carefully evaluated [69]. ACM mainly follows an autosomal dominant pattern of inheritance, with incomplete and age-related penetrance as well as polymorphic phenotypic expression [70,71]. Autosomal recessive forms have also been reported, although in a smaller number of cases (Naxos disease, caused by a deletion in the *JUP* gene, and Carvajal syndrome, caused by mutations in the *DSP* gene) [72,73,74]. In addition, alterations in number of copies (Copy Number Variation, CNV) were also associated with ACM [75].

Despite these recent advances, only in about the 50% of ACM patients a pathogenic variant is found [76,77]. However, even when it is found, the patient could show no phenotype because of the variable expressivity and incomplete penetrance [78]. Therefore, clinical translation should be done carefully, after a comprehensive personalized interpretation of all the obtained data.

## 4. Dilated Cardiomyopathy

**Figure 5:**
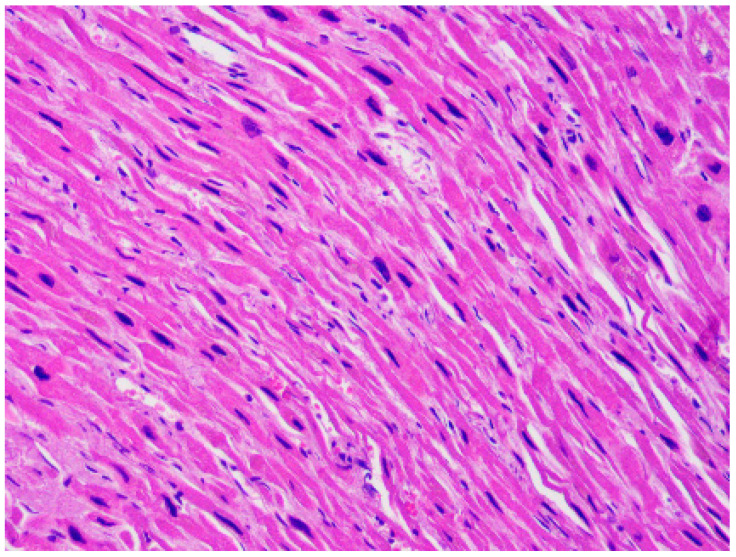
Mild fibrosis and increased nuclear-cytoplasmic ratio in the cardiomyocytes of the heart of a 45-year-old woman affected by dilated cardiomyopathy (hematoxylin and eosin stain, 40× magnification).

Dilated Cardiomyopathy (DCM) has a prevalence of 1/2500 [79]. Its mortality rate is up to 20% [80]. Death is usually due to heart failure or ventricular arrhythmias. SCD has an incidence of about 2–3%, can be the first manifestation of disease and is caused by electromechanical dissociation or arrhythmias [81]. LV dilatation and contractile impairment are the main risk factors for sudden death [82]. Greater left atrial volume also increases the risk of adverse outcomes [83]. In the paediatric population, the predictors of SD are the age at diagnosis, familiarity, and severe LV systolic disfunction [81]. At the forensic autopsies, DCM is macroscopically characterised by the dilatation of the cardiac chambers (greater in the ventricles than in the atria). These signs can be associated with other common findings, like intracavitary thrombi and, in case of right heart failure, hepatomegaly, ascites and peripheral oedema [79]. At the histopathologic examination, diffuse fibrosis with some areas of necrosis and atrophied and/or hypertrophied cardiomyocytes is usually found (Figure 5). Fibrosis plays an important role in this disease since it causes contractile impairment and ventricular re-entrant arrhythmias [84]. In DCM, two kinds of fibrosis can be found: interstitial and replacement fibrosis. Fibrosis results from the so-called “replacement”, which consists of myocyte cell death and scarring formation or directly from an expansion of interstitial collagen [85]. Replacement fibrosis is of great clinical significance because it is associated with sustained or inducible VT [86]. On the other hand, interstitial fibrosis, that is almost always found in DCM cases, is thought to cause focal tachycardias and to be involved in the maintenance of re-entry circuits [87].

### 4.1. Differential Diagnosis

**Figure 6:**
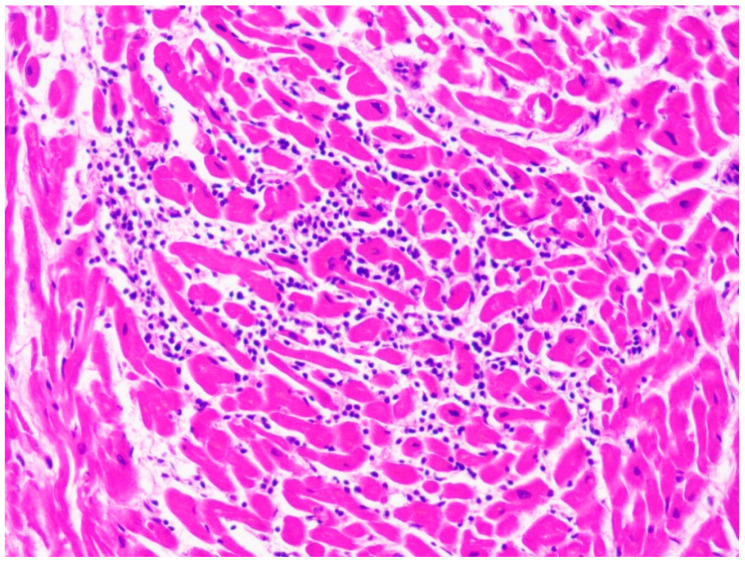
Lymphocytic infiltrate in the case of a 10-year-old child affected by myocarditis (hematoxylin and eosin stain, 40× magnification).

DCM has many possible causes. It is usually distinguished in primary (congenital) and secondary (acquired). Secondary DCM can be caused by many factors, like toxic substances (e.g., cocaine or a chronic alcohol intake > 80 g/day), pathogens (virus, bacteria, fungi, spirochete, protozoans, rickettsia), endocrine or metabolic disfunctions (electrolyte disturbances, Cushing’s disease), inflammatory conditions, and autoimmune or neuromuscular diseases [81]. It is important to note that DCM can be also a long-term toxic manifestation: for example, it can occur even 10 years after chronic anthracycline exposure [81]. Therefore, when DCM is found at the autopsy, the main tool for differential diagnosis is a complete and accurate anamnesis. From a macroscopic point of view, at the autopsy it can be difficult to differentiate DCM from ischemic cardiomyopathy, hypertensive heart disease, athlete’s heart, and other cardiomyopathies [82]. In particular, the presence of significant coronary stenosis allows to distinguish ischemic cardiomyopathy from DCM. Instead, the differential diagnosis between DCM and left ventricular noncompaction is performed through the identification of LV trabeculations, deep intertrabecular recesses, and a thin layer of normal myocardium. Histopathological examination can be of great help to distinguish a DCM from a viral or immune-mediated myocarditis thanks to the identification of a lymphocytic infiltrate (Figure 6) and, in case of infective myocarditis, the post-mortem microbiological testing (PCR) [81].

### 4.2. Genetics

As already said, nearly the 60% of familial DCM cases show genetic alteration in one of over 60 genes associated with DCM [88]. Most of the familial DCM cases are due to pathogenic variants that follow an autosomal pattern of inheritance. These pathogenic variants have been identified in several genes encoding proteins with different functions, such as ion channels, transcription factors, and sarcomeric/desmosomal/nuclear proteins. Currently, few DCM cases following an autosomal recessive pattern of inheritance have been reported [89]. Recent studies reported pathogenic variants in the *TTN* gene as the main causes of familial DCM [90]. Despite nearly the 30–35% of families affected by DCM show alterations in this gene, most of the found variants are classified as of unknown significance [90]. The second most prevalent gene in familial DCM is *LMNA*, responsible for nearly 10–15% of cases [6]. Several other genes have been associated with this disease, being responsible for nearly the 5–10% of all the familial DCM cases. Finally, other genetic alterations such as Copy Number Variation (CNV) have been reported to (rarely) be causes of DCM.

## 5. Molecular Autopsy

**Figure 7:**
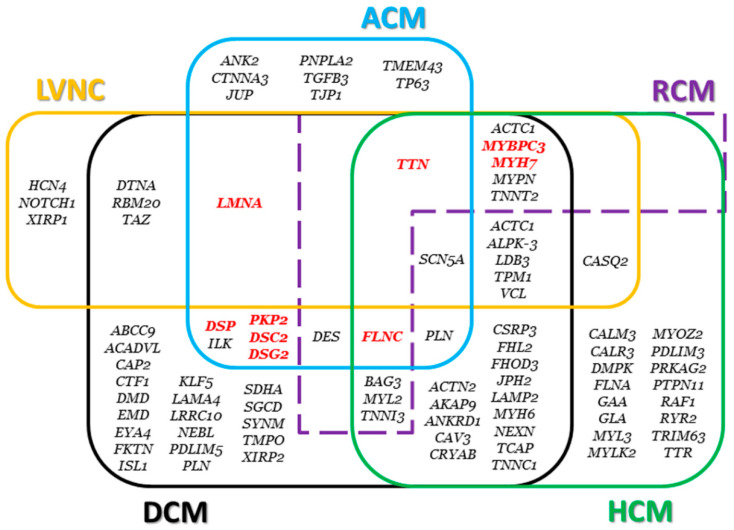
Genes associated with cardiomyopathies. The main genes are highlighted in red. Genes associated with restrictive cardiomyopathy (RCM) and left ventricular noncompaction (LVNC) are also displayed.

Molecular autopsy has been recommended as part of the autopsy process, for example, by the Heart Rhythm Society and the European Heart Rhythm Association (HRS/EHRA) [91], the European Society of Cardiology [92], the Canadian Cardiovascular Society/Canadian Heart Rhythm Society [93], and the Swiss Society of Legal Medicine [94]. Recently, the Trans-Tasman Response Against Sudden Death in the Young (TRAGADY), together with the Royal College of Pathologists of Australasia and the National Heart Foundation of New Zealand, have proposed a guide to standardize the autopsies in young cases of SCD (http://www.rcpa.edu.au/Library/Publications/Joint-and-Third-Party-Guidelines) (accessed on 3 April 2021). Despite these recent advances, these guidelines still only recommend the analysis of the main genes associated with arrhythmogenic syndromes, mainly because of economic reasons. Thanks to the recent advances in the field of genetics, more than 100 genes (about 60 genes associated with cardiomyopathies and about 40 genes associated with channelopathies) are currently known (Figure 7) [95]. The pathogenic genetic variants have been discovered thanks to the Next Generation Sequencing (NGS), that allows massive analysis of genes in a short time and in a cost-effective way [96]. Recent studies reported that the yield of genetic testing in SCD cases with autopsy findings suggestive of a cardiomyopathy is comparable with the yield in alive patients affected by cardiomyopathies [96,97]. Despite this evidence, as said, performing molecular autopsy (when indicated) is still discretionary.

## 6. Discussion

Inherited cardiomyopathies (Table 1) are relatively common and SCD is often the first manifestation of disease. When a correct diagnosis has not been made before the death, identifying a cardiomyopathy at the autopsy is extremely important for forensic and public health issues. From a public health point of view, diagnosing inherited cardiomyopathies is essential to identify other carriers of the pathogenic variants within the family and promptly adopt preventive measures. Indeed, it is very common that in the family of the victim a post-mortem diagnosis of cardiomyopathy allows new diagnoses—that would not have been otherwise made (since, as said, cardiomyopathies are generally autosomal dominant and have incomplete penetrance and variable expressivity). From a medico-legal point of view, the pathologist is often asked to determine whether the cause of the death was a condition that, for example, the cardiologist of the victim should have diagnosed. This issue is particularly relevant in countries, like Italy, where athletes have to regularly undergo cardiologic evaluation to exclude diseases, like cardiomyopathies, that contraindicate physical activity [98]. In these cases, especially when ECGs of uncertain significance were obtained and no radiological procedures were indicated, finding that the death was caused, for example, by a phenotype-positive cardiomyopathy is essential to prove the liability of the physician. Moreover, as said, it is important to distinguish inherited cardiomyopathies from myocarditis and infective cardiomyopathies. This issue is of great medico-legal relevance because, in case of death of an inpatient, if a hospital-acquired infection is suspected, it is important to assess whether the cause of the death was a primary cardiomyopathy or a myocarditis/secondary cardiomyopathy. Currently, this problem is particularly relevant, since in about one third of the critically ill COVID-19 patients a cardiomyopathy is found [99,100].

### 6.1. Importance of Good Practice

It is fundamental to carefully investigate any aspect of a SCD case (Table 1). Complete autopsies must be performed. Dissection and sampling of the heart must be done by well-trained pathologists in order to collect all the data useful for a correct diagnosis [48,54,101]. Inadequate sampling or interpretation of the microscopic features [54,82,102,103] can easily lead to missed diagnosis or misdiagnosis, especially in cases with mild phenotypes (children). Acquiring information on the clinical history of both the SD victim and his relatives is often necessary to distinguish cardiomyopathies from their many differential diagnoses [54,81,82,104] or, in case of negative autopsy, to correctly assess the significance of a variant of a gene involved in a cardiomyopathy. It is important to carefully study the clinical history of the victim and the circumstances of his death [102,104,105,106] also because, in case of negative autopsy, the pathologist can promptly suspect an IAS and thus collect fresh blood and samples of tissues for genetic testing [6,12,50,54,88,101]. This procedure is important because the DNA extracted from formalin-fixed paraffin-embedded samples is often low-template.

### 6.2. Virtopsy

In forensic practice, virtopsy (post-mortem CT and MR) has been proposed in combination with complete (or minimally invasive) autopsy, or even to replace it when the family opposes it for psycho-emotional or religious reasons [108,109]. As stated by Femia et al., in cases of SCD, virtopsy can play a strategical role in identifying the phenotype of a cardiomyopathy, in differentiating cardiomyopathies from other conditions (e.g., the asymmetric hypertrophy of HCM from a post-mortem myocardial oedema) and in reliably excluding any heart anomaly (thus orienting towards the indication to molecular autopsy) [107]. According to current evidence, both post-mortem CT and MR are accurate for the diagnosis of cardiomyopathies, but in those younger than 35 years MR is superior to CT, while, according to the current (few) evidence, in older SCD cases the concordance of post-mortem CT to conventional autopsy is higher [107]. Since ACM is characterized by a tissue replacement, virtopsy can be particularly useful when this cardiomyopathy is suspected. Kimura et al. proposed some criteria for the diagnosis of clinical cases of ACM at CT: “dilatation of the RV (RV body and outflow tract), abundant epicardial fat, myocardial fat in the RV trabeculae and moderator band, conspicuous trabeculae, and scalloped or bulging appearance of the RV free wall” [52]. Virtopsy is not only important for the diagnosis of ACM but also for distinguishing it from other conditions. For example, in up to the 62% of the CT-scans of patients with a history of myocardial infarction myocardial fat can be observed in the left ventricle [52]. This fatty infiltration tends to be thin and located in the subendocardial areas, near the occluded coronary artery, thus helping to distinguish a post-ischemic modification from a sign of ACM [52].

### 6.3. Molecular Autopsy

As said, cardiomyopathies often show mild or ambiguous macroscopic/microscopic signs of disease. This issue mainly affects young population, in which, at the same time, most of the SCDs are caused by cardiomyopathies. When, after the autopsy, the pathologist cannot make inference on the cause of the death, a molecular autopsy (post-mortem genetic testing) should be performed. This procedure is not mandatory, but it is highly recommended, since in cardiomyopathies the diagnostic rate is relatively good (80% for HCM, 50% for ACM, 30–40% for DCM) [6,12,88]. It is important to remark that in the last years rare variants in genes that code for structural proteins have been described as possible inducers of heart arrhythmias in absence of structural alterations [110]. This is because genes can affect the expression of nearby or distant genes, and some of these variants have recently been associated with the development of cardiac pathologies [111]. Moreover, as said, structural anomalies tend to be progressive and may cause arrhythmias in the young even before the complete development of the phenotype [112,113,114]. Proper sampling and storage of the blood and/or of the tissues (liver, spleen and/or heart) for the genetic testing is crucial for optimizing the results of the procedure. The choice of the source of DNA (blood vs tissues) depends on the protocol that the forensic geneticist wants to adopt. At the same time, high-throughput techniques (NGS) are particularly recommended, since variants of many different genes can cause inherited cardiomyopathies [6].

Currently, the main challenge in this field is represented by the clinical interpretation of the NGS findings. The significance of the variants is assessed following clinical guidelines, in particular those proposed by the American College of Medical Genetics and Genomics (ACMG) and the Association for Molecular Pathology (AMP) [115,116,117]. In the forensic field, the assessment of the significance of the variants is mainly limited by two issues: the absence of forensic guidelines (that take into account the peculiarities and the limits recurring in forensic practice) and the fact that most of the variants cannot be interpreted because of the lack of the necessary data. Indeed, in a forensic investigation acquiring complete information on the clinical history of the victim (and of his family) and performing a segregation analysis must be authorized by the competent authorities and can be extremely time-consuming (because of the bureaucratic procedures) [6]. Moreover, since relatively few molecular autopsies are performed, the lack of shared data can limit the interpretation of a variant.

Another important issue is represented by the fact that, since the significance of a variant depends on the data known at the time of the investigation, it can change over time.

In 2020, Campuzano et al. analyzed how many variants of genes involved in IAS had to be reclassified after 10 years [118]. The authors found that the 69.23% and the 94.11% of the variants found in, respectively, clinical and forensic cases had to be reclassified and that many of the variants previously classified as likely pathogenic were re-classified as variants of uncertain significance (VUS). In total, the prevalence of VUS was 18.75% in 2010 and 60.15% in 2020. Regarding the cardiomyopathies, while the prevalence of VUS in HCM and DCM cases did not change after reclassification, in ACM cases it significantly increased. The authors noted that the increase of the VUS prevalence in IAS cases depended on the higher stringency of the diagnostic criteria of the ACMG/AMP guidelines.

In 2021 Vallverdù-Prats et al. reevaluated 39 rare variants of genes associated with ACM that had already been classified according to the ACMG/AMP guidelines in 2016 [119]. In particular, the authors reported a 17.95% decrease in VUS and a 5.12% increase in likely pathogenic variants [119]. They underlined that these variations were mainly due to the updated global frequencies, thus underlying the importance of periodic reevaluations.

Hence, the assessment of the significance of a variant is an extremely critical step in the forensic evaluation of a SCD case. For instance, in forensic field, finding only a variant of uncertain significance means failing to find the cause of the death and thus making impossible to draw medico-legal conclusions on a specific case, while, on the other hand, misinterpreting a variant (e.g., a variant is classified as likely pathogenic and then, after ten years, is re-classified as VUS) can severely jeopardize the reliability of a medico-legal report. The communication of the results of a molecular autopsy to the competent authorities and, if requested by the local law, to the family of the victim should stress that the significance of a variant can be “dynamic”, largely depending on the data that are available at the time of the investigation, and that, if not certain, should be periodically reevaluated. As said, it can be particularly complex to explain the (absence of) diagnostic value of a VUS, especially when it is the only finding in an autopsy-negative forensic case.

## 7. Conclusions

Autopsies of forensic cases of inherited cardiomyopathies are extremely challenging when the phenotypes of these diseases are mild or difficult to be distinguished from physiological or other pathological conditions. In these cases, if accurate microscopic analysis is not diriment, virtopsy and (in particular) post-mortem genetic testing can be of great help. The results of molecular autopsies should always be interpreted by a team composed by (at least) a forensic pathologist and a forensic geneticist and, if uncertain, should be always communicated stressing that the significance of a variant can be dynamic.

## Figures and Tables

**Table 1:** The main features of inherited HCM, Arrhythmogenic Cardiomyopathy (ACM) and Dilated Cardiomyopathy (DCM).

	HCM	ACM	DCM
Prevalence	1/500 [12]	1/2000 [51]	1/2500 [79]
Typical macroscopic features	Abnormal wall thickness (≥15 mm) of the LV that cannot be explained by abnormal loading conditions [48]	Fibrofatty replacement of the myocardium of the right ventricle and/or the left ventricle [54]	LV or biventricular dilatation that cannot be explained by abnormal loading conditions or coronary artery disease [101]
Typical histopathological features	Myocytes hypertrophy, disarray, thickened intramural arterioles with luminal narrowing, myocardial fibrosis [102,103]	Fibrofatty replacement of the ventricular myocardium with a subepicardial-mid-mural or transmural distribution [54]	Replacement fibrosis, interstitial fibrosis, atrophied and/or hypertrophied cardiomyocytes, nuclear pleomorphism [82]
Differential diagnosis (examples)	Athlete’s heart, hypertensive cardiomyopathy, glycogen/lysosomal storage diseases, amyloid/sarcoid cardiomyopathy [104]	*Adipositas cordis,* Uhl’s anomaly, PRKAG2 cardiac syndrome, myocarditis, HCM [54]	Acquired DCMs, ischemic cardiomyopathy, hypertensive heart disease, athlete’s heart, left ventricular noncompaction [81,82]
Main SCD risk factors	LV wall thickness ≥ 30 mm, anamnestic factors (personal history of cardiac arrest, sustained ventricular arrhythmias, syncope), familiarity for SCD, ejection fraction < 50%, nonsustained ventricular tachycardia (NSVT), LV apical aneurysm, extensive late gadolinium enhancement, mutations of troponin T gene [102,104]	Number of premature ventricular complexes and of anterior and inferior leads with T-wave inversion at 24-h Holter monitoring, younger age, male sex [105]	Mutations of *LMNA* gene, personal history of syncopes or nonsustained ventricular tachycardia, delayed enhancement identification, frequent premature ventricular contractions, familiarity for SCD [106]
Genetics	More than 50 genes coding for sarcomeric proteins (e.g., *MYH7, MYBPC3, TNNI3, TNNT2, TPM1, MYL3*) and non-sarcomeric proteins (e.g., *CSRP3, FHL1, PLN*) [50]	More than 15 genes coding for desmosomal proteins (e.g., *PKP2, DSP, DSG2, DSC2*) and non-desmosomal proteins (e.g., *TMEM43, PLN*) [54]	More than 60 genes coding for proteins with different functions, such as ion channels, transcription factors, sarcomeric/desmosomal/nuclear proteins (e.g., *TTN, LMNA, MYH7, TNNT2, MYBPC3, RBM20, MYPN, SCN5A, BAG3, PLN*) [101]
Diagnostic rate of post-mortem genetic testing	Nearly 80% [6,12]	Nearly 50% [6,12]	Nearly 30–40% [6,12,88]
Indicated technique for virtopsy	MR (technically difficult) [107]	MR [107]	CT, MR [107]

## Data Availability

Not applicable.
